# Efficacy and safety of fire needle vs conventional acupuncture in the treatment of postherpetic neuralgia: A protocol for systematic review and meta analysis

**DOI:** 10.1097/MD.0000000000022563

**Published:** 2020-10-09

**Authors:** Lunbin Lu, Jun Xiong, Zhijun Chen, Xingchen Zhou, Jun Chen, Genhua Tang, Siyuan Zhu, Zhiying Zhong, Han Guo

**Affiliations:** a Jiangxi University of Traditional Chinese Medicine; b The Affiliated Hospital of Jiangxi University of Traditional Chinese Medicine, Nanchang, China.

**Keywords:** efficacy evaluation, fire needle, meta-analysis of protocols, postherpetic neuralgia, randomized controlled trials

## Abstract

**Background:**

Fire needle therapy has the double function of acupuncture and moxibustion, which has both the stimulation of needle and the warm stimulation of moxibustion. As an important part of acupuncture and moxibustion, fire needle has been widely used in clinical treatment since ancient times in China. Postherpetic neuralgia (PHN) is a kind of chronic and solid neuropathic pain with persistent and intense pain after the skin lesion of sores has been completely eliminated. The clinical treatment of PHN is mostly integrated therapy. In recent years, many literatures have reported that the curative effect of fire needle on PHN is accurate. The purpose of this protocol is to describe how to accumulate evidence for further understanding of the status quo and reliability of clinical practice in the treatment of PHN with fire needle.

**Methods:**

Seven electronic databases were used to retrieve the literature for the PHN randomized controlled trials, including 3 English databases (PubMed, EMBASE, the Cochrane Central Register of Controlled Trials [Cochrane Library]) and 4 Chinesedatabases (Chinese National Knowledge Infrastructure, Chinese VIP Information, Wanfang Database, and Chinese Biomedical Literature Database). This systematic review will include all randomized controlled clinical trials using fireneedle therapy for PHN. Pain intensity, safety and cost, quality of life, global perceptionare outcomes. The selection of the study will be completed independently by 2 reviewers, extract the data, and evaluate the quality of the study before selecting the title, abstract, and full text. Revman 5.4 software will be used to perform meta-analyses of randomized controlled trials, where risk ratios for dichotomous data and standardized or weighted mean differences for continuous data are the results.

**Result:**

**Conclusion:**

This proposed systematic review will provide up-to-date evidence to assess the effect of fire needle for patients with PHN.

**Registration:**

INPLASY202080029.

## 1 Introduction

Postherpetic neuralgia (PHN) is a kind of chronic and solid neuropathic pain with persistent and intense pain after the skin lesion of sores has been completely eliminated.^[1]^ Most scholars believe that the local residual pain longer than 1 month to 6 months after complete regression of skin lesions can be defined as PHN.^[2–5]^ The clinical manifestations of the pain are varied, such as continuous or paroxysmal pain, induced or spontaneous pain, burning or throbbing pain, and a series of skin paresthesia, delayed sensation, hypersensitivity, and so on.^[1]^ Although age, pain severity in the acute stage of sores, severity of rashes, psychological factors, diabetes, nutritional status, and immune status may all be associated with the onset of PHN, the risk factors for the accurate prediction of PHN have not been determined.^[6–10]^ Studies have shown that the most relevant risk factor is age. The incidence rate of PHN increases sharply with age. Nearly 10% of patients over 40 years old develop PHN, while 20% to 50% of patients over 60 years old develop PHN.^[11]^ As a result of chronic pain, patients may experience depression, inattention, fatigue, insomnia, and reduced daily activities and social interactions that reduce the patients quality of life.^[12]^

Due to persistent or intermittent spontaneous pain, PHN severely affects the patient's daily activities (such as dressing, bathing, sleeping), quality of life, general health, mental health (such as depression and difficulty concentrating), and social and economic well-being.^[13,14]^ Although little is known about the pathophysiology of PHN, autopsies of patients with PHN have revealed demyelination and axonal loss of peripheral nerves and sensory roots^[15]^

Current treatments for PHN include antiepileptics (such as gabapentin and Pregabalin), tricyclic antidepressants, lidocaine, and opioid receptor agonists.

Although there are various treatments for PHN, there is still no consensus on its effectiveness due to the difficulty of treating PHN.^[1]^ Systematic reviews have shown that there is not enough evidence to support lidocaine or antidepressants as a first-line treatment for PHN.^[1,16]^ Tricyclic antidepressants may cause adverse events, including sedation, dry mouth, psychosis, rhythm disturbances, and weight gain. In addition, antiepileptic drugs, lidocaine and opioids will bring dizziness, saliva, dizziness, fatigue, walking instability, and other adverse events.^[17–20]^

Fire needle therapy is a treatment method that makes a thick and thin needle made of a special material burn red on the fire and then quickly stab into certain points and parts of the human body.^[21]^ The fire needle therapy has a long history and has become an important part of Chinese medicine as early as the Huangdi Neijing Era.^[22]^ Three filiform needles will be used to tie them together, taking into account the patients endurance. The position and depth of the needle insertion is determined by our own experience.^[23-15]^^[23–25]^ The role of the fire needle has to enhance immunity, regulate blood circulation, prevent diseases. In some clinical trials, fire needle has been shown to be effective against PHN.^[26]^ In addition, the fire needle is safe and reliable, easy to operation, non-toxic side effects. In spite of this, the mechanism of fire needle for PHN has not been completely studied. However, the aim of this study is conducted to assess the efficacy and safety of fire needle for PHN. On the basis of clinical research, the findings of this review will be reliable.

## 2 Methods

### 2.1 Study registration

The protocol has been registered on the International Platform of Registered Systematic Review and Meta-analysis Protocols (INPLASY) (registration number, INPLASY202080029) basing on the Preferred Reporting Items for Systematic Reviews and Meta-Analyses Protocols (PRISMA-P) statement guidelines.

### 2.2 Inclusion criteria and exclusion criteria

#### 2.2.1 Research type

This review will include all available randomized controlled trials (RCTs) on fire needle treatment for PHN. Other studies such as case reports, reviews, retrospective studies, and studies using inappropriate random sequence generation methods will be excluded. The language limit is Chinese and English.

#### 2.2.2 Participant type

Participants with a diagnosis of PHN who had sustained pain for more than 3 months will be all focused on. Age, gender, course of disease, and source of cases are not limited.

#### 2.2.3 Intervention measures

This review aims to investigate the clinical effect of fire needle on PHN. It will be included in the experimental study of the use of fire needle. Studies will be excluded if the efficacy of fire needles in combination with other treatments is not clear. The therapeutic intervention of controlled group can be conventional acupuncture, electro-acupuncture, auriculo-acupuncture, or pharmcological therapy.

#### 2.2.4 Major research indicators

##### 2.2.4.1 Primary outcome

Studies which applied scales such as the Visual Analogue Scale (VAS), Numerical Rating Scale (NRS), Verbal Rating Scale (VRS), the Faces Pain Scale-Revised (FPS-R), etc. that were used to measure the intensity of pain will be included.

##### 2.2.4.2 Secondary outcomes

1. Global impression (the proportion of participants whose symptoms improved after treatments);
2. Quality of life;
3. Safety as measured by the incidence and severity of adverse effects;
4. Costs.

### 2.3 Search methods for study identification

#### 2.3.1 The following databases will be searched:

1. The Cochrane Skin Group Trials Register (the inception to 2020.8);
2. MEDLINE (the inception to 2020.8);
3. EMBASE (the inception to 2020.8);
4. The Cochrane Central Register of Controlled Trials (CENTRAL; the inception to 2020.8);
5. Chinese Biomedical Literature Database (CBM; the inception to 2020.8);
6. Chinese Medical Current Content (CMCC; the inception to 2020.8);
7. China National Knowledge Infrastructure (CNKI; the inception to 2020.8).

The following search terms will be used: post herpetic neuralgia, PHN, herpes zoster, shingles and fire needle, electro-acupuncture, fire needle, pyonex, moxibustion, pricking blood, three-edged needle. This study will use this strategy to search all the above databases. There are no language or type of publication restrictions. The search strategy for MEDLINE can be found in Table 1. Searching other resources. A review or meta-analysis of relevant RCT systems will be conducted via electronic search.

**Table 1:**
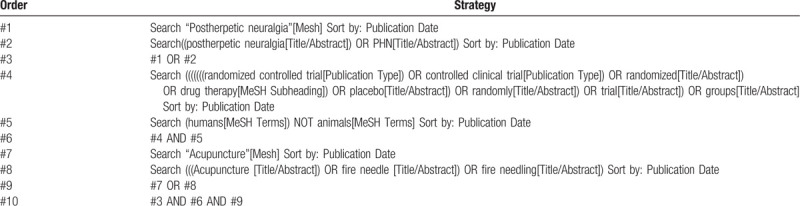
The search strategy for Pubmed.

### 2.4 Data collection and analysis

#### 2.4.1 Study selection

We will select an RCT that compares the efficacy and safety of fire needle therapy for PHN. Articles that meet 1 of the following criteria will be excluded:

1. the duplicates,
2. the participants did not meet the diagnosis criteria of PHN or the diagnosis criteria is unknown,
3. not RCT studies,
4. the studies in which the experimental participants don’t receive a combination of fire needle therapy and conventional therapy as the main intervention.
5. the intervention includes any other traditional Chinese medicine (TCM) therapy
6. incomplete data which will be needed.

**Figure 1:**
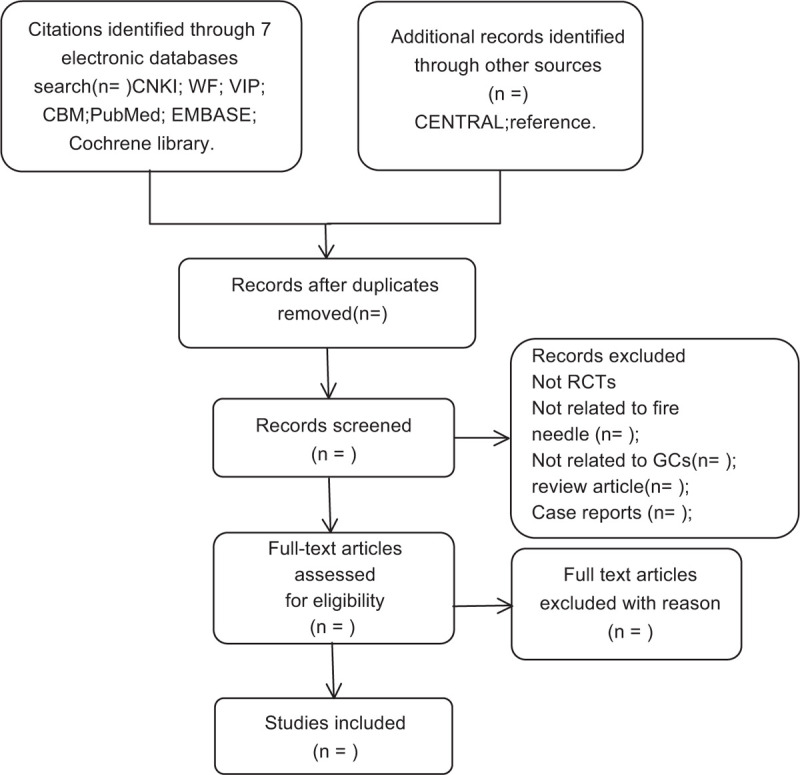
Flowchart of literature selection.

Two authors will assess whether these studies meet the criteria. We will discuss and resolve any objections contained in the articles. The specific process of studies screening will be displayed in a Preferred Reporting Items for Systematic Reviews and Meta-Analyses (PRISMA) flow diagram (Fig. 1).

#### 2.4.2 Data extraction and management

The data will be extracted independently by the 2 authors (ZYZ and JC). Any differences that arise during the data extraction process will be discussed and resolved. We will extract the following information: participant details (baseline data, diagnostic criteria), study details (authors, country, year of publication, multicenter or single center study), the interventions and the outcomes (ADL score, VAS score, ROM, and adverse events incidence), the methods used (registry platform, sample size, blinding method). Unreported data will be obtained by contacting the corresponding author through us.

#### 2.4.3 Assessment of risk of bias in included studies

The risk of bias will be assessed by 2 reviewers (ZYZ and HG) with the Cochrane Collaborations tool for risk of bias assessment. The risk of bias in included studies will be evaluated according to the following aspects: sequence generation, allocation sequence concealment, blinding of participants and personnel and outcome assessors, incomplete outcome data, selective outcome reporting, and other sources of bias. The assessments will be classified into 3 levels: low risk, high risk, and unclear risk.

#### 2.4.4 Measures of treatment effect

RevMan V.5.4 will be used for data analysis and quantitative data synthesis. For continuous data, we will use standard mean difference (SMD) to measure the treatment effect with 95% confidence intervals (CIs). For dichotomous data, a risk ratio (RR) with 95% CIs for analysis will be adopted.

#### 2.4.5 Dealing with missing data

The corresponding author will be contacted by telephone or e-mail for insufficient or missing trial data. If the missing data cannot be supplied or failure to contact the author, a limited analysis will be performed which based on the available data and we will discuss the potential impact of the missing data.

#### 2.4.6 Assessment of heterogeneity

On the basis of the data analysis, random effect or fixed effect models will be employed according to the heterogeneity given by *I*^*2*^ statistic value. To be concrete, a fixed effect model will be adopted if the heterogeneity is indicated as high (*I*^*2*^ < 50%); otherwise, a random effect model will be applied on the contrary.

#### 2.4.7 Assessment of reporting bias

Funnel plot will be used to assess reporting biases of the studies include. We will consider that the reporting bias is existing and the reliability is low if the points on both sides of the funnel plot are dispersed and asymmetrical. Conversely, if the points on either side of the funnel plot are symmetrically distributed in substantial, we will consider the reporting bias as non-existent and the result is reliable.

#### 2.4.8 Data synthesis and subgroup analysis

We will use RevMan software (V5.4, The Nordic Cochrane Centre, The Cochrane Collaboration, Copenhagen, Denmark) to conduct all analyses. And we will select a random effects model or fixed effects model to merge the primary and secondary outcome indicators in accordance with the results of heterogeneity test. We will apply the fixed effects model for data synthesis of low heterogeneity (*I*^2^ < 50%) while the random effects model will be conducted if the heterogeneity is significant (*I*^2^ ≥ 50%). It is considered that differences are statistically significant if the results of Z test show that *P* value is less than .05, and the 95% CI dose not contain 0 (for continuous variables) or the 95% CI dose not contain 1 (for dichotomous variables). If heterogeneity is evaluated as significant (*I*^2^ ≥ 50%) and the trials included are adequate, we will perform a subgroup analysis to explore the potential source of the heterogeneity according to the difference in participant characteristics, interventions, controls, and outcome measures.

#### 2.4.9 Sensitivity analysis

We will carried out sensitivity analysis to identify the quality and robustness of the meta analysis result when the outcome analyses involve a large degree of heterogeneity, according to sample size, methodological quality, and the effect of missing data.

#### 2.4.10 Grading the quality of evidence

We will evaluate the quality of evidence and rate it into 4 levels: high, moderate, low, or very low in accordance with the Recommendations Assessment, Development and Evaluation (GRADE) guidelines.

#### 2.4.11 Ethics and dissemination

Ethical approval will not be necessary because the data included in our study are derived from published literature and are not linked to individual patient data. The systematic review providing implication of the effectiveness and safety of fire needle for PHN will be published in a peer reviewed journal or conference presentations.

## 3 Discussion

Postherpetic neuralgia (PHN) is a kind of chronic and solid neuropathic pain with persistent and intense pain after the skin lesion of sores has been completely eliminated.^[1]^ Most scholars believe that the local residual pain longer than 1 month to 6 months after complete regression of skin lesions can be defined as PHN.^[1–4]^ As a result of PHN, patients daily activities, quality of life, general health, mental health, and social and economic well-being are affected.^[27]^ Although the first line of treatment for PHN is antiepileptics (such as gabapentin and Pregabalin), tricyclic antidepressants, lidocaine, and opioid receptor agonists, the therapeutic effect is not very ideal. Ancient Chinese medicine has a certain understanding of herpes zoster, and there are Chinese medicine and acupuncture and moxibustion and other different treatment methods.^[28]^ At present, the clinical results show that the fire needle has a significant effect on the treatment of PHN.^[29]^ In China, it is recommended to use the fire needle to treat PHN, but there is no comprehensive evidence of high authors contribution quality. The evaluation of this literature shows that the operation of fire needle in the treatment of postherpetic neuralgia is safe and effective, and the cure rate and effective rate of fire needle therapy, especially in combination with other therapies, are obviously superior to other therapies. However, this study may have limitations that might limit its ability to generate conclusions based on high confidence. Specifically, there may be significant heterogeneity in the forms of fire needle used and the qualities of methodology. There will also most likely be differences in outcomes measured and tools used. Inherent uncertainty exists by pooling these data within constructed domains. More electronic databases or gray studies were not searched in this systematic review, which may affect the retrieval and results of randomized controlled trials. This review is registered on INPLASY (registration number, INPLASY202080029, https://inplasy.com/?s=INPLASY202080029).

## Author contributions

All authors have read and approved the publication of the protocol.

**Conceptualization:** Lunbin Lu, Jun Xiong.

**Data curation:** Lunbin Lu, Jun Xiong, Lunbin Lu, Jun Chen, Genhua Tang, Siyuan Zhu, Zhiying Zhong, Han Guo.

**Formal analysis:** Jun Xiong, Lunbin Lu.

**Investigation:** Jun Xiong, Lunbin Lu.

**Methodology:** Lunbin Lu, Xingchen Zhou, Jun Chen.

**Software:** Jun Xiong, Siyuan Zhu.

**Supervision:** Jun Xiong.

**Writing – original draft:** Jun Xiong, Lunbin Lu, Xingchen Zhou, Han Guo.

**Writing – review and editing:** Jun Xiong, Zhiying Zhong, Jun Chen.

## Abbreviations

Abbreviations: ADL = ability assessment of daily living activities, CBM = Chinese Biomedical Literature Database, CBM = Chinese Biomedical Literature, CI = confidence intervals, CMCC = Chinese Medical Current Content, CNKI = Chinese National Knowledge Infrastructure, GRADE = Recommendations Assessment, PHN = postherpetic neuralgia, PRISMA-P = Preferred Reporting Items for Systematic Reviews and Meta-Analyses Protocols, RCTs = randomized controlled trials, RR = rate ratio, TCM = traditional Chinese medicine, International Clinical Trials Registry Platform, VAS = visual analogue scale, VZV = varicella-zoster virus.
